# Interdisciplinary insights on the future of food systems research: perspectives from the next generation of research leaders

**DOI:** 10.1017/S1368980022001641

**Published:** 2022-11

**Authors:** Cherie Russell, Ashley Schram, Libby Salmon, Amy Carrad, Liza Barbour, Jennifer Lacy-Nichols, Oliver Huse, Priscila Machado, Joshua Gilbert, Christina Zorbas, Courtney Thompson

**Affiliations:** 1 School of Exercise and Nutrition Sciences, Deakin University, Geelong, VIC 3220, Australia; 2 School of Regulation and Global Governance, Australian National University, Canberra, ACT, Australia; 3 School of Medical, Indigenous and Health Sciences, Faculty of Science, Medicine and Health, University of Wollongong, Wollongong, NSW, Australia; 4 Department of Nutrition, Dietetics & Food, Monash University, Notting Hill, VIC, Australia; 5 Centre for Health Policy, Melbourne School of Population and Global Health, University of Melbourne, Carlton, Melbourne, VIC, Australia; 6 Global Obesity Centre, Institute for Health Transformation, Faculty of Health, Deakin University, Geelong, VIC, Australia; 7 Institute for Physical Activity and Nutrition, Deakin University, Geelong, VIC, Australia; 8 Jumbunna Institute for Indigenous Education, University of Technology, Sydney, NSW, Australia; 9 Charles Sturt University, Wagga Wagga, NSW, Australia; 10 Queensland University of Technology (QUT), Faculty of Health, School of Exercise and Nutrition Sciences, Kelvin Grove, QLD, Australia

**Keywords:** Food systems, Food systems governance, Sustainable food systems, Sustainable healthy diets, Food sovereignty, Food equity, Early career researcher

## Abstract

Our dominant food system is a primary driver of worsening human and planetary health. Held in March 2022, the Public Health Association of Australia’s Food Futures Conference was an opportunity for people working across the food system to connect and advocate for a comprehensive, intersectoral, whole-of-society food and nutrition policy in Australia to attenuate these issues. Conference themes included food systems for local and global good; ecological nutrition; social mobilisation for planetary and public good; food sovereignty and food equity. Students and young professionals are integral in transforming food systems, yet they are under-represented in the academic workforce, across publishing, scientific societies and conference plenaries. A satellite event was held to platform initiatives from early career researchers (ECR) in areas integral for improving planetary and public good. The research topics discussed in this commentary reflect sub-themes of the conference under investigation by ECR: food systems governance and regulation; local food policies; commercial determinants of health; sustainable healthy diets; and food equity and sovereignty.

Our current consumptogenic food system has exceeded planetary boundaries and is a primary driver of deteriorating human and planetary health^(1)^. Held in March 2022, the Public Health Association of Australia’s Food Futures Conference was an opportunity for those working across the food system to connect, share their work and advocate for a sustainable, equitable and healthy food system. The conference theme, ‘transforming food systems for the planetary and public good’, consisted of five sub-themes: (1) food systems for local and global good, (2) ecological nutrition, (3) social mobilisation for planetary and public good, (4) food sovereignty and (5) food equity. Students and young professionals, as future leaders, are integral in transforming food systems. However, given the scarcity of funding available for research, academics early in their careers have reduced opportunities to contribute to research and thus are under-represented in grants, publishing, scientific societies and conference plenaries^(2)^.

Thus, we developed the satellite event *The Future of Food Research: An Early Career Showcase* to platform initiatives from early career researchers (ECR) in areas integral for improving planetary and public good. The showcase presented valuable opportunities for capacity development, networking and reflections from ECR about how to navigate the challenges of building an engaged academic career. This commentary synthesises the valuable, internationally applicable work and perspectives of ten emerging food systems leaders to highlight their voice and opinions and to demonstrate the value of including ECR in all aspects of conducting and disseminating research. This includes fostering leadership, developing intersectoral partnerships and building workforce capacity. This work describes key food systems considerations and highlights implications for future research, policy and practice in this space.

## Body

### Food systems governance and regulation

Healthy and equitable food systems transformation – wherein the systems of food production through to food consumption contribute to a safe, sustainable, affordable, accessible and nutritious food supply – requires widespread transformation in the policies and processes governing food systems^(3)^. ECR have been pioneering research into the impacts of international trade and investment agreements on national food environments – making a significant contribution to our understanding of the impact of public policy on nutrition and health. This work demonstrates that the current trade and investment system has produced (and reproduced) a global food system that preferences heavily processed products and increasing corporate concentration^(4–7)^. Intersections with contemporary issues such as calls for reduced animal products in the global food supply have also been highlighted. For example, trade and investment rules regarding non-discrimination may inhibit national policies which attempt to obstruct the marketing of alternative plant-based proteins (e.g. restrictions on monikers such as ‘sausage’ or ‘burger’) at the request of domestic meat industries^(7)^. Greater engagement from public health nutritionists in this space is required to ensure that trade and investment agreements can be leveraged to deliver healthy and equitable food systems.

Breast-feeding is the optimal or ‘first food system’^(8)^ for infants. When a mother cannot provide her own milk, the WHO^(9)^ recommends donor human milk, obtained from a milk bank or through safe informal arrangements in the community. Various milk-sharing practices and policies address concerns about risk^(10)^ and ethics^(11)^ but face commercial pressures from novel technologies and international trade^(12)^, potentially distorting infant feeding systems. By drawing theories of ‘multicentric governance’^(13)^, empirical studies of legal structures and interviews of key actors (mothers, milk banks, health professionals and policy makers), research demonstrates that, in Australia, the regulation of milk sharing is fragmented, with conflicts between policy and social objectives and sources of authority. Policy focused on milk as a product and milk banking lacked integration with breast-feeding, while informal systems of milk sharing captured local social processes important to resilient infant feeding systems globally.

### Local food policy

In research and public fora on food system governance, much attention is given to the role of state and federal governments, and the food industry. However, relatively little is known about how local governments and civil society organisations contribute to creating a healthy, sustainable and equitable food system. The Strengthening Local Food Systems Governance project included a policy mapping study that audited food system-related policies developed by all local governments in the Australian states of New South Wales and Victoria (*n* 207)^(14)^. To expand on these findings, focus groups with six local governments identified common enablers of and barriers to development and implementation of these policies (e.g. funding, collaboration, legislative mandate). Additionally, a survey of civil society organisations revealed a wide variety of organisations, activities and policy priorities, which were further explored through focus groups with nine organisations. To strengthen the role of local governments and civil society organisations in food systems, they require dedicated funding to undertake food systems work and better coordination across all levels of government, between government departments and across all sectors of society. This research provides evidence to support advocacy for food and nutrition-related legislation that protects and promotes health.

Complementary research has explored the policy response of local government authorities globally to contribute to the population-wide shift towards healthy and sustainable diets called for by the EAT-Lancet Commission^(1)^. Based on a review^(15)^ of relevant United Nations’ publications, a set of thirteen desirable diet-related practices were identified and presented to demonstrate how they can trigger a whole-of-system transformation, including (i) where food is sourced, (ii) what is eaten and (iii) how food is consumed. To explore and map policy options available to local government authorities to facilitate the population-wide uptake of these practices, a scoping review^(16)^ of Milan Urban Food Policy Pact signatory cities was published, demonstrating bold leadership and innovation occurring by these urban cities. This review highlighted that while a holistic approach, considering health, equity and the wide scope of the food supply chain is being taken, opportunities exist to leverage the dual benefits to human and planetary health of policy actions, such as those which discourage the overconsumption of food, including animal-derived foods, and the regulation of ultra-processed foods.

### Commercial determinants of health

National dietary guidelines are one important lever to promote healthy and sustainable food systems and diets, yet research suggests that few dietary guidelines present straight-forward advice regarding ultra-processed foods, instead reverting to euphemisms that can be exploited by food companies for marketing^(17)^. Understanding the gap between evidence and policy is a political question. Research has been undertaken to explore the intersection between corporations, politics and health – a field referred to as the Commercial Determinants of Health – and questions who has power, where power comes from, how power is exercised and how to challenge power^(18)^. Applying a Commercial Determinants of Health lens to food system governance questions the logic of public–private partnerships and the risks of conflicts of interest when powerful food companies have a seat at the table with nation states^(19)^. Ultimately, efforts to promote more sustainable, healthy and equitable food systems must contend with the vested interests that pull the financial strings and drive ever-growing corporate consolidation^(20)^.

The food and beverage industry frequently acts to influence food and nutrition policies, preventing the introduction of barriers to the sale of their unhealthy products^(21)^. Research is underway to describe the commercial determinants of health in the Philippines, and how this disrupts policy development. The food and beverage industry in the Philippines builds its power and influence by occupying key positions, forming coalitions, operationalising its extensive resources and swaying constituents to support industry objectives. Industry engages in a range of tactics aimed at influencing policy development and implementation, including contacting policy makers directly, promoting ‘substitute’ policies, presenting evidence and data that they have generated themselves and offering gifts and financial incentives to government agencies and individuals^(22,23)^. Industry messaging commonly frames globally recommended policies as having unintended negative impacts and being ineffective at improving health. As a result, food and nutrition policies in the Philippines have been delayed, watered-down or abandoned. As such, ECR-led research is important for advocating for legislation to protect and promote health through food and nutrition policies.

### Sustainable healthy diets

Measuring and monitoring characteristics of diets at global and national levels are needed to inform and assess the effectiveness of policy actions that promote sustainable healthy diets. In 2019, the FAO of the United Nations and the WHO reported sixteen guiding principles for sustainable healthy diets, but these principles have not yet been operationalised into a diet quality metric^(24)^. Novel research aimed to develop a multidimensional diet quality index based on recommendations for sustainable healthy diets through a scoping review of the literature on how principles of such diets are considered in metrics used to assess diet quality globally. No existing dietary metric captures all principles of sustainable healthy diets. Notably, the significance of ultra-processing, environmental and cultural aspects of diets is generally understated. This highlights the importance of revising current dietary recommendations, especially to include emerging topics.

Food systems monitoring has identified consumer behaviours as key drivers of the food system, which can be addressed to encourage more healthful, sustainable diets by individuals, communities or nations^(25–29)^. However, to date, there have been no reported measures for assessing food acquisition, preparation, meal practices and storage: all key components of food literacy^(26,30)^. The development of a measure to assess this component of the food system has been hindered by low agreement on a definition and conceptualisation of food literacy. However, a recent publication reported agreement on the Vidgen & Gallegos^(31)^ model as the core conceptualisation of food literacy^(32)^. This allowed for the development of the International Food Literacy Questionnaire (IFLQ-19), a questionnaire which adheres to the four domains and eleven components of food literacy by Vidgen & Gallegos^(31)^ using comprehensive validation techniques. The IFLQ-19 fills a substantial gap by allowing for consumer behaviour monitoring and surveillance within the food system.

### Food equity and sovereignty

There is little literature in Australia that acknowledges the ingenuity and adaptability of Indigenous people to Western agriculture, with the conversation instead contending whether agriculture systems were evident before colonisation^(33,34)^. There are only a few instances which discuss the co-existence of Indigenous and non-Indigenous People^(35)^, and even less acknowledgement that considers the cross learning of cultures on stations. The Australian perspective is approximately 40 years behind the discussion when acknowledging international perspectives^(36)^, creating further tension and unease between non-Indigenous and Indigenous People. Emerging research aims to reform the current position in Australia by enabling Indigenous culture (and therefore Indigenous agriculture) to be seen as a fluid concept, rather than having a static demeanour. Ultimately, this work contends that an Aboriginal person farming today is as authentically Aboriginal as what this person would have been throughout time – we are the same people, but just a people in change.

With global food systems currently dominated by powerful actors, fair opportunities to participate in food and health decision-making have been compromised^(37,38)^. Indeed, the voices and values of those experiencing social and/or economic disadvantage, and the poorest diet-related health, remain under-represented in food system decision-making, policies, research and advocacy efforts^(39,40)^. To challenge similar exclusion practices, international movements by disability and First Nations advocates have long championed the phrase ‘Nothing About Us Without Us’^(41,42)^. If we are to ensure that everyone has access to a healthy diet, especially in the wake of the global COVID-19 pandemic, we must start listening to and acting on the voices of communities who are facing the harshest impacts of our unhealthy, unfair and unsustainable food systems^(38)^. Irrespective of location, this is likely to necessitate the creation of safe spaces for people to share their stories (supported by culturally appropriate communication), shared ownership over public food system initiatives (compared with tokenistic involvement of the public) and ongoing investment in diversifying and empowering community leaders in our food systems^(43)^.

## Conclusion

ECR are integral in food systems transformation and advocacy. Improved representation and diversification of ECRs across food systems research is important; both because diversity in voice and opinion are essential for equitable practice, but also to foster leadership skills and innovation into the future. Indeed, recent reforms from the National Health and Medical Research Council have sought to lower the barriers for ECR to access competitive funding^(44)^. Overall, this commentary highlights the diverse, yet interconnected work guiding future research and practice related to food policies and systems governance at local, national and international levels; the influence of commercial factors and the need to maintain food sovereignty and equity as central foci. This work highlights how ECR are integral in upholding the legacy of leaders in this field through publication contributions and by building workforce capacity. These themes, and the inclusion of researchers across all career stages in publishing, scientific societies and conference plenaries, must be prioritised and acted upon if we are to ensure continuity in progression of food systems transformation for healthy and sustainable food systems for all.
